# Black soldier fly larvae: a one health approach to investigate gut, and organ health and meat quality response in slow-growing chickens

**DOI:** 10.1186/s12917-024-04394-0

**Published:** 2024-12-27

**Authors:** Edoardo Fiorilla, Ilario Ferrocino, Marta Gariglio, Francesco Gai, Valeria Zambotto, Laura Ozella, Irene Franciosa, Marzia Giribaldi, Sara Antoniazzi, Federica Raspa, Eleonora Erika Cappone, Dmitri Fabrikov, Sara Pozzo, Valentina Bongiorno, Alice Calissano, Dorotea Ippolito, Stefania Bergagna, Karthika Srikanthithasan, Claudio Forte, Maria Teresa Capucchio, Achille Schiavone

**Affiliations:** 1 Department of Veterinary Sciences, University of Turin, Grugliasco, Italy; 2 Department of Agricultural, Forest and Food Sciences, University of Turin, Grugliasco, Italy; 3 Institute of Sciences of Food Production, National Research Council, Grugliasco, Italy; 4 Department of Biology and Geology, University of Almería, Almería, Spain; 5 Department of Food Safety, Nutrition and Veterinary Public Health, Italian National Institute of Health, Rome, Italy; 6 Veterinary Medical Research Institute for Piedmont, Liguria and Aosta Valley, Turin, Italy

**Keywords:** *Hermetia illucens*, Agro-ecology, Local breeds, Microbiota

## Abstract

**Background:**

The inclusion of sustainable protein sources in poultry feed has become essential for improving animal welfare in livestock production. Black soldier fly larvae are a promising solution due to their high protein content and sustainable production. However, most research has focused on fast-growing poultry breeds, while the effects on native breeds, such as the Bianca di Saluzzo, are less explored. This study aimed to evaluate the impact of BSFL supplementation in the diet of slow-growing chickens, with a focus on growth, intestinal health and meat quality for final consumers.

**Results:**

The study demonstrated that Black soldier fly larvae supplementation, both in dehydrated and live form, improved growth performance, with an increase in final weights compared to the control group. No significant differences were found in feed conversion ratio, liver and spleen weight or histomorphometry between groups. Black soldier fly larvae supplementation did not negatively affect immune function or liver metabolism. Higher production propionic acid was detected in the black soldier fly larvae fed groups compared to the control, suggesting an effect on volatile fatty acid production. Gut microbiota analysis showed an increase in beneficial bacteria, such as Faecalibacterium, in the live larvae group. Furthermore, the meat fatty acid profile and atherogenicity and thrombogenicity indices did not undergo significant changes, implying a low potential risk to consumers’ cardiovascular health.

**Conclusions:**

Black soldier fly larvae supplementation in the diet of Bianca di Saluzzo improved growth without compromising animal health or meat quality. Moreover, the increase in beneficial volatile fatty acids and the modulation of gut microbiota suggest a positive impact on gut health. Finally, the absence of negative effects on meat lipid profiles confirms nutritional safety for consumers, making black soldier fly larvae a valid alternative in poultry feed. These results offer new perspectives for the use of Black soldier fly larvae in the nutrition of local breeds, contributing to sustainability in line with the One Health approach.

## Background

In the last decades, the livestock sector has faced growing challenges related to animal welfare [1], food safety [2] and environmental sustainability [3] stimulating research in improving these areas of livestock production. These efforts have led to the exploration of alternative protein sources like Black Soldier Fly Larvae (BSFL), which are promising for chicken nutrition due to their high protein content, and ability to convert organic waste into valuable biomass [4]. BSFL can thrive on various organic substrates, including food waste, supporting waste management and nutrient recycling. Their role in the circular economy is significant, contributing to protein production, reducing greenhouse gas emissions, and improving soil health through their digestion by-products [5]. Overall, this integration in poultry feed as meal or as whole larvae as the case of this study aligns with environmental goals by promoting waste reduction and resource efficiency [6].

Most studies recently have mainly focused on commercial fast-growing hybrids, while the impact of insect-based feed, specifically live or dehydrated larvae, on indigenous poultry breeds remains less explored [7]. These indigenous breeds represent a valuable genetic and cultural resource and understanding how they can benefit from the integration of BSFL could open new perspectives for the sustainability of livestock farming, especially in rural and organic farming [8]. In this context, revitalizing agroecology with local, slow-growing chicken breeds involves integrating ecological principles into sustainable poultry farming to enhance biodiversity, animal welfare, and local economies, promoting the use of natural processes and local resources, which aligns with the traits of slow-growing and indigenous chicken breeds that are well-adapted to their environments and require minimal inputs [9]. These indigenous chickens play a key socio-economic role in rural areas, offering low-risk, low-labor production, making them ideal for agroecological systems [10]. They are often more resilient to local conditions, having adapted to various agro-climatic challenges through natural selection. The conservation of genetic diversity in poultry, as seen in Italy and Turkey, is vital for preserving the unique traits of local breeds [11]. Integrating these breeds into small-scale production, as demonstrated in Italy, can open niche markets for high-quality poultry products, supporting local economies and biodiversity conservation [12, 13]. Overall, revitalizing agroecology with local chicken breeds requires a holistic approach combining conservation, sustainable breeding, and market development to build resilient food systems that benefit both people and the environment. This strategy not only supports ecological sustainability but also fosters social equity and community-driven economic development, aligning with agroecology’s broader goals of restoring ecosystem services and improving human welfare [14].

The concept of One Health, which recognizes the interconnection between human health, animal health and environmental health perfectly fits into this context, because indigenous breeds, thanks to their ability to adapt could play a key role in promoting environmentally and consumer friendly agriculture [15]. Moreover, in many rural communities, these breeds also represent a source of livelihood and income, contributing to local food security. Their integration between agroecology and the one health approach fits perfectly into a resilient agricultural system, ensuring benefits for animals, ecosystems and the people who depend on them [16, 17]. Among the indigenous Italian poultry breeds, the Bianca di Saluzzo deserves special attention for its unique characteristics and the historical and cultural value it represents. This breed is characterized by completely white plumage, yellow legs and high-quality meat, highly appreciated for its intense flavor and tender consistency [16]. The Bianca di Saluzzo is a slow-growing breed, which adapts perfectly to extensive, free-range farming systems. In the past, it was a fundamental element for local farming communities, but with the advent of commercial hybrids, its presence has drastically reduced [18]. Thanks to the interest in quality poultry production and the rediscovery of local culinary traditions, in recent years several recovery projects have been launched to preserve this breed, enhancing it as an example of excellence in the Italian agri-food heritage [19]. The ability of the Bianca di Saluzzo to adapt to diets based on local resources makes it particularly interesting in terms of sustainability [20]. The integration of BSFL larvae into the diet of this breed could represent a promising solution to improve growth performance without compromising meat quality, while maintaining the nutritional values ​​that make it so appreciated.

Considering these premises, this study aims to evaluate the impact of incorporating both dehydrated and live BSFL into the diets of slow-growing indigenous chickens, specifically focusing on the Bianca di Saluzzo breed. The research assesses growth performance, feed efficiency, meat quality, and gut health, adopting a holistic approach based in One Health and agroecological principles with an intermediate slaughter at 147 days to assess potential age-related effects. By addressing this gap in the existing literature, the study seeks to enhance understanding of BSFL’s role in chicken nutrition while supporting the conservation of indigenous breeds and promoting resilient agricultural practices.

## Methods

### Birds, husbandry, diets and black soldier fly supplementation

The research was conducted at the University of Turin’s poultry facility in northwestern Italy, with approval from the University’s Bioethical Committee (Approval No. 814715). Detailed information on the experimental design is available in Fiorilla et al. [21], as this study continues the same project and used the same birds. To support the use of local genetic resources and advance conservation efforts, the animals used in the trial were hatched from eggs collected at the Avian Conservation Centre for Local Genetic Resources, affiliated with the University of Turin. This center plays a key role in preserving and studying indigenous avian species, contributing to the preservation of poultry biodiversity and deepening the understanding of their distinct genetic traits.

In summary, the experiment involved 144 male Bianca di Saluzzo chickens, each 39 days old, individually marked with wing tags, and housed in the experimental facility. There were six replicates of eight birds for each of the three treatments. The study lasted 135 days, ending when the birds reached 174 days of age, with an intermediate slaughter at 147 days to assess potential age-related effects. Before each slaughter, individual birds were weighed. Two birds per replicate were selected based on average live weight, resulting in a total of 36 birds per slaughter.

The chickens were slaughtered following European Regulation (EC) No 1099/2009, which ensures animals are treated humanely during the process. They were first electrically stunned to make them unconscious, then bled out, as required by the regulation.

Three dietary treatments were tested: the control group (C) received a basal diet adapted to niche poultry market, using local ingredients (maize meal 46.1%, field bean 11.0%, pea protein 10.8%, barley 4.7%, sunflower meal 9.5%, maize gluten 11.6%, soybean oil 1.6%; apparent metabolizable energy 11.8 MJ/kg, crude protein 18.0%, ether extract 3.7%, crude fiber 4.8%) (Table 1) [20]; the three experimental groups were given the same basal diet with an addition of 5% of the expected daily dry matter intake, either as dehydrated (DL) (Table 1) or live (LL) black soldier fly larvae (Table 1). Mortality and health status were monitored daily throughout the study. From day 39, the birds’ live weight and feed intake were recorded every 21 days, and the feed conversion ratio (FCR) was calculated. As noted earlier, slaughters were conducted at 147 and 174 days of age. The day before each slaughter, all birds were weighed individually, and two birds per pen (24 birds per slaughter) were selected based on their average live weight. After a 12-hour fasting period, the selected birds were reweighed to determine their slaughter weight (SW), then electrically stunned and exsanguinated in compliance with EU regulations (Council Regulation (EC) No 1099/2009 of 24 September 2009). The carcasses were plucked, eviscerated, and weighed, with the ready-to-cook carcass (RTCC) weight recorded [21]. The spleen and liver were also weighed post-evisceration.

**Table 1:** Analyzed composition of the basal diet (C), the dehydrated (DL) and live black soldier fly larvae (LL) fed to an indigenous chicken breed over the period 39–174 days of age

	C	DL	LL
Chemical composition (g/100 g feed)			
Dry matter	90.3	93.9	33.9
Crude protein	18.1	33.9	43.3
Ether extract	3.63	31.4	16.1
Crude fiber	4.80	7.98	9.09
AME (MJ/kg)	11.9	17.7	14.3
Fatty acids (%)			
C12:0	0.12	33.2	43.6
C14:0	0.10	7.75	8.54
C16:0	12.9	16.1	12.4
C17:1	0.05	0.83	0.26
C18:0	2.89	3.09	2.56
C18:2n6	51.7	13.5	7.59
C18:3n3	3.74	1.25	0.99
C20:2n6	0.08	0.17	0.16
C20:3n6	0.18	0.03	0.02
C20:4n6	0.03	0.04	0.01
SFA	15.9	60.8	68.1
MUFA	26.9	15.1	16.0
PUFA	55.7	14.9	8.76
Aminoacidic composition (g/100 g crude protein)			
Alanine	6.53	3.10	3.73
Arginine	6.53	1.83	2.52
Aspartic Acid	9.80	3.54	4.41
Glutamic Acid	17.4	3.87	5.46
Glycine	8.17	2.41	2.98
Histidine	2.45	0.99	1.94
Isoleucine	4.19	1.70	2.12
Leucine	8.17	2.65	3.10
Lysine	6.53	2.63	3.36
Methionine	2.12	0.76	0.88
Phenylalanine	5.12	1.71	1.97
Proline	6.53	2.04	2.73
Serine	4.79	1.53	2.05
Threonine	3.76	1.50	1.92
Tyrosine	3.10	2.67	3.21
Valine	4.79	2.44	2.97

*SFA *saturated fatty acids, *MUFA* mono-unsaturated fatty acids, *PUFA* poly-unsaturated fatty acids, *AME* apparent metabolizable energy

### Histomorphological investigations and blood immune markers

At 147 and 174 days of age, samples of gut segments approximately 5 cm in length were collected from the intestines of 12 animals in each group and flushed with 0.9% saline solution to remove residual content. In particular, the duodenal loop, the section before Meckel’s diverticulum (jejunum), was sampled. Additionally, specimens from the spleen (whole organ) and the left lobe of the liver (0.5–1.5 g/organ) were obtained. Samples were preserved in 10% buffered formalin, embedded in paraffin wax blocks, sectioned into 5-µm slices, mounted on glass slides, and stained with haematoxylin and eosin (H&E) for histomorphological examination. For the morphometric analysis one slide for each organ was evaluated by a light microscope (Zeiss Axiophot microscope, Carl Zeiss, Oberkochen, Germany) at a magnification of 2.5x. Pictures of the intestinal segments were taken with a Nikon DS-Fi1 digital camera (Nikon Corporation, Minato, Tokyo, Japan) and morphometric analysis was performed using ImageJ software (6.0 version, Media Cybernetics, MD, USA) and the image processing package Fiji. Specifically, 10 well-oriented and intact villi and 10 crypts from the jejunum were measured for each bird [22]. The morphometric indices evaluated included villus height (from the tip of the villus to the crypt), crypts depth (from the base of the villus to the submucosa), and their ratio [23] Additionally, the widths of the villi and of the mucosal and muscular layers were measured. Villi area was measured using the formula (2π)(Villus width/2)(Villus height).

Histopathological changes in the gut, spleen and liver were examined, and a semiquantitative scoring system was used to evaluate inflammatory and degenerative changes, rating them as absent (score = 0), mild (score = 1), moderate (score = 2), or severe (score = 3). Three independent examiners assessed all slides, resolving any discrepancies through collective examination using a multi-head microscope until a consensus was reached. Blood samples were collected at slaughter, and serum was separated by centrifugation at 3000 rpm for 10 min. Concentrations of IgM (pg/mL), IgA (ng/mL), IgG (ng/mL) and IL-6 (pg/mL) were determined using enzyme-linked immunosorbent assays (ELISA), specific for each analyte. All samples were analyzed in triplicate to ensure the accuracy of the results.

### Liver metabolism

Liver samples taken at 174 days of age (*n* = 12) were immediately frozen at −80 °C after collection. The tissues were homogenized in a 0.1 M TRIS/HCl buffer solution containing 0.1 mM EDTA and 0.1% (v/v) Triton X-100, then centrifuged at 20,000 × g for 30 min at 4 °C. The resulting supernatant was stored at −80 °C. The activities of liver metabolic enzymes, including alanine aminotransferase (ALT; EC 2.6.1.2), aspartate aminotransferase (AST; EC 2.6.1.1), glutamate dehydrogenase (GDH; EC 1.4.1.2), pyruvate kinase (PK; EC 2.7.1.40), glucose 6-phosphate dehydrogenase (G6PDH; EC 1.1.1.49), and fructose 1,6-bisphosphatase (FPBase; EC 3.1.3.11), were measured according to Bis anger [24]. Absorbance was measured using a 340 nm spectrophotometer (Power Wavex microplate). Enzyme activity was expressed as the amount of enzyme needed to oxidize or reduce 1 µmol of NADH/NADP per minute per milligram of protein at 37 °C.

### Microbiota

Caecum content samples were collected from chickens at 174 days of age (*n* = 12) using sterile equipment to prevent contamination. These samples were then immediately frozen at −80 °C for storage until further analysis. Total DNA was extracted from the caecal samples of 48 Bianca di Saluzzo chickens and analyzed using a metataxonomic approach to identify potential variations in microbiota composition. The 16 S rRNA gene, specifically targeting the V3-V4 regions, was amplified, purified, tagged, and pooled according to Illumina’s guidelines [25]. The raw files (fastq) containing 250-bp paired-end reads generated by the Illumina MiSeq platform with V2 chemistry were processed using QIIME 2 software [26]. Following the methodology described by Callahan et al. [27], the Cutadapt tool was used to remove primer sequences, and the DADA2 algorithm was applied for denoising the reads through the q2-dada2 plugin within the QIIME 2 environment. Taxonomy classification was performed using the QIIME 2 feature-classifier against the SILVA database. To improve the reliability of the sequence reads, amplicon sequence variants (ASVs) with a read count of less than five in at least two samples were excluded.

### Intestinal volatile fatty acids

The procedure used to collect cecum content for microbiota analysis was also employed to obtain aliquots for volatile fatty acid (VFA) analysis at 147 and 174 days of age. The quantification of VFAs was adapted from the method described by Guantario et al. [28]. For this, 300 mg of caecal samples were suspended in 600 µL of 0.1 N H_2_SO_4_ solution and vortexed. The mixture was then centrifuged at 15,000 x g for 10 min at 4 °C. The resulting supernatant was transferred into 2 mL clear glass vials (Sigma-Aldrich).

The VFA analysis was conducted using High-Performance Liquid Chromatography (HPLC) with a Dionex UVD 340U UV/VIS Detector (Thermo Fisher), equipped with a 300 × 7.8 mm Aminex HPX-87 H column (Bio-Rad) and a guard column. A 30 µL sample was injected and isocratically separated in 0.005 N H_2_SO_4_ at a flow rate of 0.6 mL/min at 41 °C. The VFAs were detected by UV light at 210 nm and identified using an external standard curve, which was created with standards dissolved in 0.1 N H_2_SO4 and covering the following ranges: 4.95–148.5 mg/100 mL succinic acid; 9–270 mg/100 mL lactic acid; 10.5–314.4 mg/100 mL acetic acid; 9.85–285.5 mg/100 mL propionic acid; 9.4–282.1 mg/100 mL butyric acid; 9.5–285.1 mg/100 mL isobutyric acid; 9.1–273.4 mg/100 mL iso-valeric acid; 9.1–273.2 mg/100 mL valeric acid; and 4.95–148.5 mg/100 mL citric acid.

### Feed, black soldier fly larvae and breast meat fatty acid profile, atherogenicity and thrombogenicity indices

The fatty acid profiles of BSFL, diets, and breast fillet were analyzed using the method described by O’Fallon et al. [29]. In brief, 500 mg of freeze-dried meat, 140 mg of larvae, or 800 mg of poultry diet were placed into a 25 ml glass tube, to which 5.3 ml of methanol and 0.7 ml of 10 M KOH in aqueous solution were added. The tubes were incubated in a water bath at 55 °C for 1.5 h, then cooled to room temperature in a bath of cold tap water. After cooling, 0.58 ml of 12M H_2_SO_4_ in aqueous solution was added. The tubes were then incubated again at 55 °C for another 1.5 h before being cooled in the same manner. Next, 3 ml of hexane was added, followed by vortex mixing for 5 min and subsequent centrifugation. From the top hexane layer, containing the extracted fatty acid methyl esters (FAME), 40 µl were removed and diluted in 1 ml of hexane for gas chromatographic analysis. The FAMEs were injected into an Agilent 7890 GC system (Palo Alto, CA) equipped with an on-column injector and a flame ionization detector. Separation was performed using a CP-Sil88 column for FAME (100 m x 0.25 mm x 0.25 μm) under the conditions described by Placha et al. [30]. FAME identification was accomplished by comparing retention times with reference standards (Supelco 37 Component FAME mix, 47885-U, Merck).

The atherogenicity (AI) and thrombogenicity (TI) indices were calculated according to Ulbricht and Southgate [31] as follows:

$$\mathrm{AI}=\frac{\left[\mathrm C12:0+\left(4\times\mathrm C14:0\right)+\mathrm C16:0\right]}{\left(\sum\mathrm{MUFA}+\sum\mathrm n-6+\sum\mathrm n-3\right)}$$

$$\mathrm{TI}=\frac{\left(\mathrm C14:0+\mathrm C16:0+\mathrm C18:0\right)}{\left(0.5\times\sum\mathrm{MUFA}\right)+\left(0.5\times\sum\mathrm n-6\right)+\left(3\times\sum\mathrm n-3\right)}$$

### Data analysis

Statistical analyses were performed using R software, Version 4.4.0. Each pen was considered as the experimental unit for the growth performance (*n* = 6 pens per treatment), while the individual bird was considered the experimental unit for the analysis of intestinal and organ health, liver metabolism and intestinal volatile fatty acids. The normality of data distribution was verified using the Shapiro–Wilk test and the homogeneity of variance was established by means of Levene’s test. Growth performance, intestinal and organ health, liver metabolism and intestinal volatile fatty acids were analyzed by fitting a general linear model (GLM). The results are expressed as the least square mean and standard error of the mean (SEM). P values *p* < 0.05 were considered statistically significant. The microbiota α-diversity index (Simpson, Shannon and Chao1) were obtained using R software using package vegan. Spearman’s rank correlation coefficient between microbial ASVs and VFAs was obtained through the function psych and plotted using the corrplot package in R (FDR < 0.05).

## Results

### Growth and slaughtering performance

Table 2 summarizes the SW and FCR values of the birds. No significant differences were observed between the three experimental treatments, except in SW, where the DL and LL groups exhibited higher weights compared to the C group. Moreover, as expected, birds achieved a higher final live weight at the older slaughter age of 174 days compared to 147 days, showing a 12% increase. Additionally, the FCR was significantly higher at 174 days, with a 13% increase compared to 147 days (*p* < 0.05). Table 2 also presents data on slaughtering performance, where no significant differences were found in the weights of the RTCC, liver, or spleen across different diets, ages, or their interaction.

**Table 2:** Slaughter weight (SW), feed conversion ratio (FCR), ready to cook carcass (RTCC) and organs of an indigenous chicken breed fed a diet supplemented with dehydrated black soldier fly larvae and live black soldier fly larvae over the periods 39–147 and 39–174 days of age (means, *n* = 6)

	Diet (D)			Age (A)		SEM	*p*-value		
	C	DL	LL	147 d	174 d		Diet	Age	D x A
SW (g)	2335^b^	2440^a^	2412^a^	2251	2541	20.60	0.029	< 0.001	0.848
FCR (g/g)	3.51	3.37	3.48	3.24	3.67	0.046	0.114	0.019	0.544
RTCC (SW %)	65.1	65.3	65.2	65.3	65.1	0.433	0.997	0.927	0.539
Spleen (SW %)	0.19	0.19	0.18	0.20	0.19	0.009	0.878	0.200	0.329
Liver (SW %)	1.55	1.56	1.57	1.53	1.59	0.060	0.975	0.354	0.655

*C* control, *DL* dehydrated larvae, *LL* live larvae, *DxA *interaction diet/age, *SEM *standard error of mean

a, b: *P*<0.05

### Intestinal morphometry and organ scores, immune-markers activity and liver metabolism

Table 3 outlines the histological scores observed in the gut and major organs. The inclusion of dehydrated and live BSFL in the diet did not significantly impact the severity of these changes in any of the organs examined. The results highlighted no notable differences between the different treatment groups. The histomorphometry of the jejunum, also shown in Table 3 reveals no significant differences in the morphometric items considered between the C, DL, and LL groups, or between birds slaughtered at 147 and 174 days of age. This suggests that neither the diet nor the age at slaughter influenced gut morphology. Additionally, blood immune-markers remained consistent across all groups, indicating that the dietary treatments did not affect immune function.

**Table 3:** The histopathological evaluation of gut, liver and spleen (score 0–3*), histomorphometry of the jejunum (mm) and the interleukin in blood plasma of an indigenous chicken breed fed a diet supplemented with dehydrated black soldier fly larvae and live black soldier fly larvae over the periods 39–147 and 39–174 days of age (means, *n* = 12)

	Diet (D)			Age (A)		SEM	*p*-value		
	C	DL	LL	147 d	174 d		Diet	Age	D x A
Gut and organs histology scores (0–3)									
Intestine	1.1	1.1	1.2	1.3	1.2	0.063	0.691	0.194	0.370
Liver	0.4	0.5	0.3	0.4	0.4	0.086	0.551	0.284	0.434
Spleen	0.07	0.1	0.2	0.1	0.1	0.027	0.432	0.434	0.271
Gut histomorphometry (mm)									
Villus heights	0.811	0.780	0.860	0.747	0.887	0.0243	0.407	0.004	0.855
Villus width	0.095	0.089	0.099	0.095	0.093	0.0053	0.137	0.669	0.405
Villus heights/Crypts depth	15.5	15.8	16.4	14.3	17.4	0.584	0.796	0.008	0.765
Crypts depth	0.054	0.051	0.053	0.053	0.052	0.0025	0.529	0.551	0.604
Mucosae width	1.32	1.31	1.38	1.25	1.43	0.0238	0.484	0.101	0.777
Muscularis layers width	0.223	0.224	0.239	0.218	0.239	0.0164	0.558	0.125	0.021
Villus Area	0.243	0.218	0.266	0.227	0.258	0.0223	0.101	0.090	0.534
Immune markers									
IgM (pg/mL)	2254	2308	2065	2171	2247	110.8	0.642	0.728	0.711
IgA (ng/ml)	1.05	1.09	1.02	1.08	1.02	0.0248	0.493	0.179	0.067
IgG (ng/ml)	1127	1190	1256	1115	1267	157.5	0.946	0.629	0.851
IL-6 (pg/ml)	53.5	46.4	39.6	50.4	42.6	4.95	0.521	0.429	0.870

*C *control, *DL *dehydrated larvae, *LL *live larvae, *DxA *interaction diet/age, *SEM* standard error of mean

*score = 0; absent, score = 1; mild, score = 2; moderate, score = 3, severe

Immune response indicators, including IgM, IgA, IgG, and IL-6, also showed no significant differences between the C, DL, and LL groups. This stability suggests that the dietary treatments did not negatively impact the birds’ immune systems. Table 4 presents the effects of the diets on liver enzymes, evaluated at 174 days of age. Most liver enzymes, such as G6PDH, ALT, AST, and GDH, showed no significant differences between the control and experimental diets. Only FBPase levels were higher in the DL and LL groups compared to the control, and PK levels were elevated in the DL group (*p* < 0.05).

**Table 4:** The hepatic metabolism enzymes of an indigenous chicken breed fed a diet supplemented with dehydrated black soldier fly larvae and live black soldier fly larvae at 174 days of age (means, *n* = 12)

	Diet (D)			SEM	*p*-value
	C	DL	LL		
FBPase	20.3^b^	30.3^a^	32.0^a^	1.22	< 0.001
PK	31.7^b^	33.8^a^	29.0^b^	0.713	0.016
G6PDH	2.81	2.90	2.03	0.302	0.416
ALT	171	172	172	5.07	0.995
AST	472	497	534	14.4	0.209
GDH	1642	1505	1412	47.9	0.155

*C *control, *DL *dehydrated larvae, *LL *live larvae, *SEM *standard error of mean, *FBPase *Fructose-1,6-bisphosphatase, *PK *Pyruvate Kinase, *G6PDH* Glucose-6-phosphate dehydrogenase, *ALT *Alanine Aminotransferase, *AST *Aspartate Aminotransferase, *GDH *Glutamate Dehydrogenase

a, b: *P*<0.05

### Microbiota

**Fig. 1:**
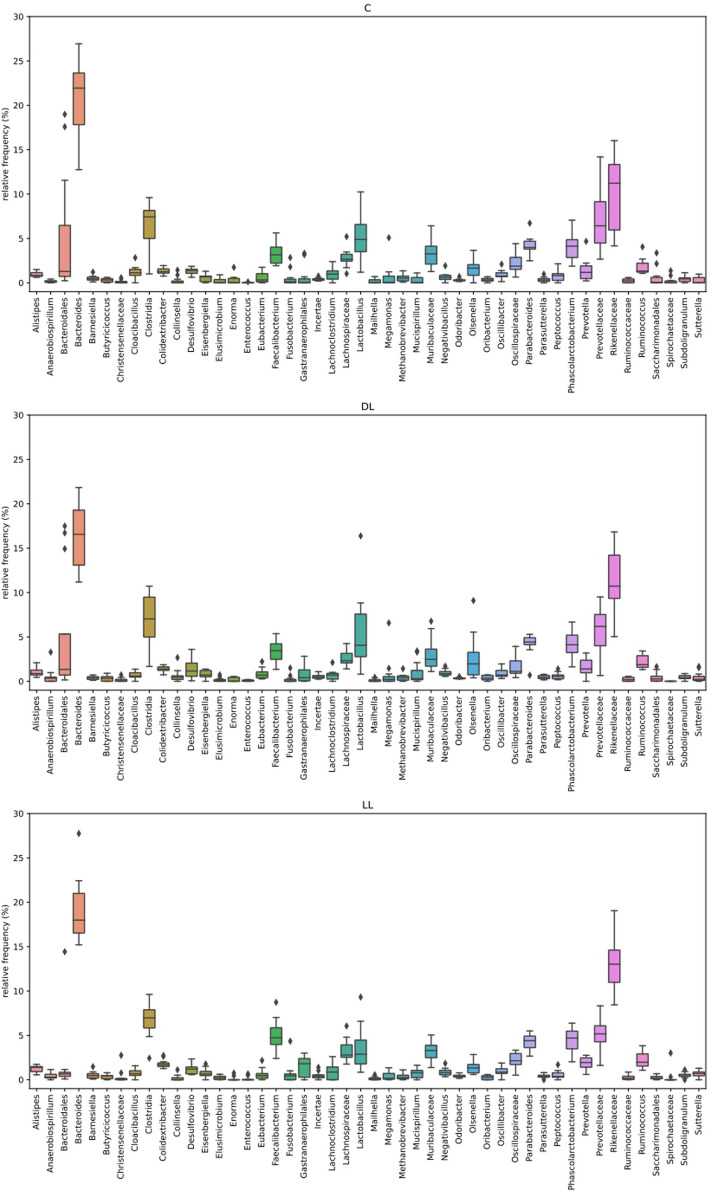
Total ASV (Amplicon Sequence Variant) present in the gut of an indigenous chicken breed fed a diet supplemented with dehydrated black soldier fly larvae and live black soldier fly larvae over the period 39–174 days of age

**Fig. 2:**
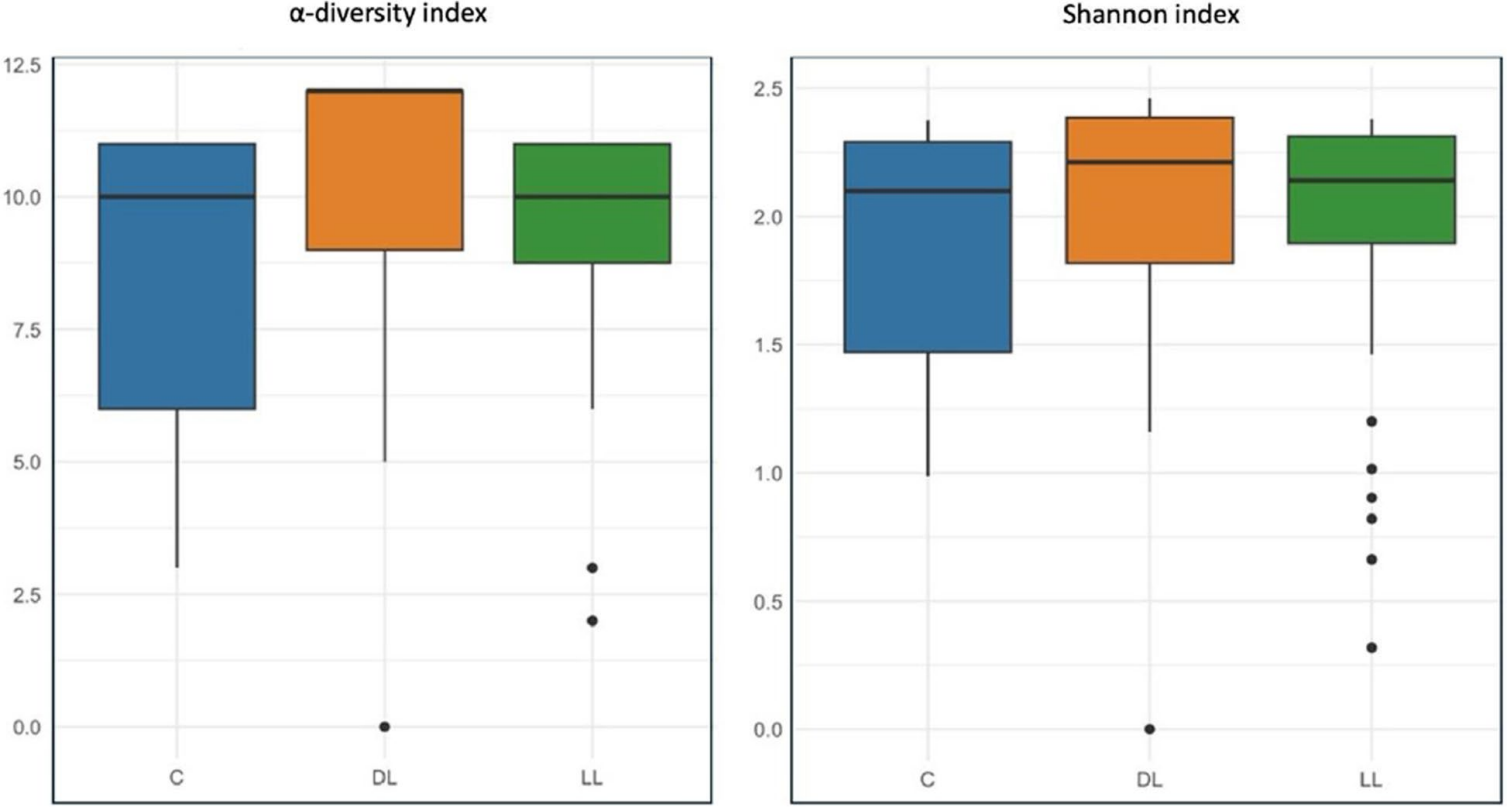
Analysis of the microbiota α-diversity and Shannon indicators in the cecal digesta of an indigenous chicken breed fed a diet supplemented with dehydrated black soldier fly larvae and live black soldier fly larvae over the period 39–174 days of age

**Fig. 3:**
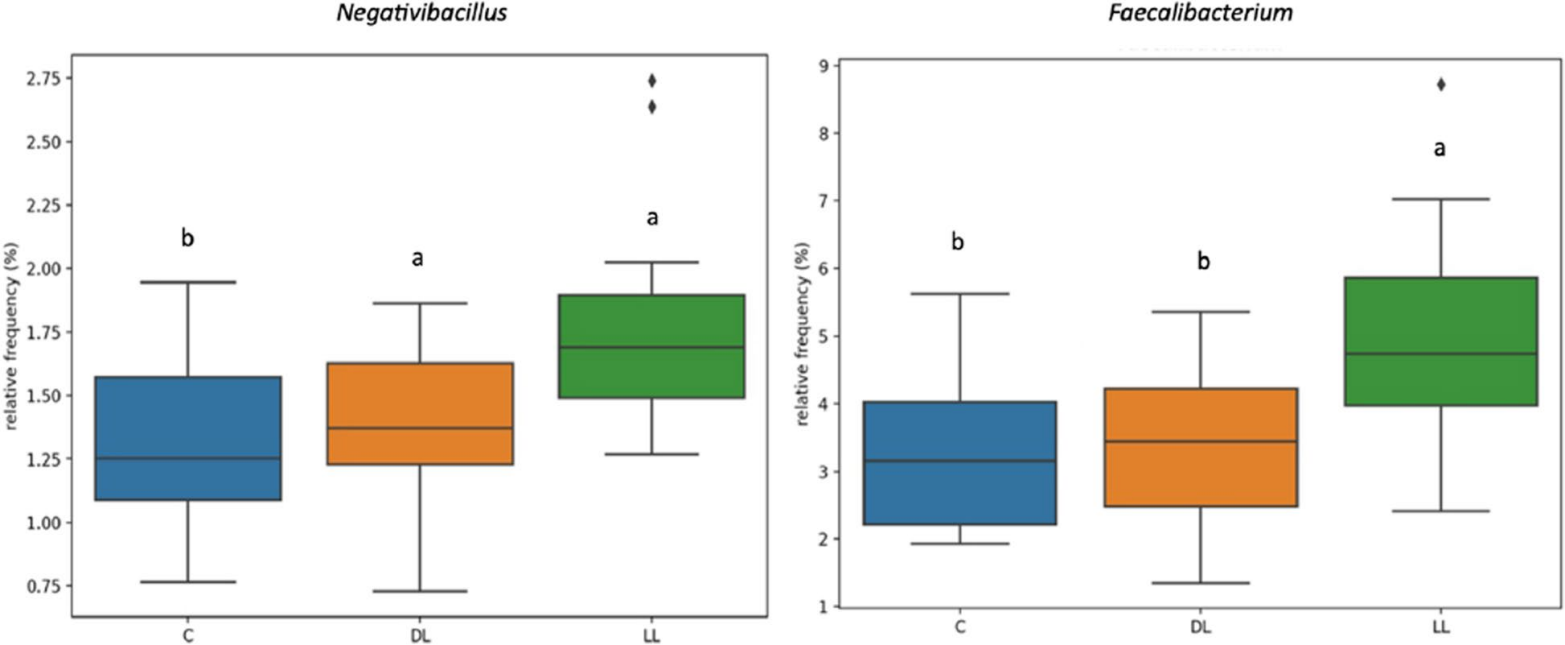
Significantly different total Amplicon Sequence Variant of bacteria present in the gut of an indigenous chicken breed fed a diet supplemented with dehydrated black soldier fly larvae and live black soldier fly larvae over the period 39–174

Figure 1 illustrates the relative concentration of ASVs in the caecum across the three dietary treatments: C, DL, and LL. The data reveal distinct variations in microbiota composition between the groups. To assess microbial diversity, α-diversity and the Shannon diversity index were calculated to quantify both richness and evenness of species within each treatment group (Fig. 2). The analysis indicated no significant differences in microbial diversity between the experimental treatments. Figure 3 presents the bacterial taxa that exhibited significant differences in concentration across the three dietary treatments. Notably, *Negativibacillus* was present in higher concentrations in both the DL and LL treatments compared to the C treatment. On the other hand, *Faecalibacterium* was found in significantly greater amounts only in the LL treatment when compared to both C and DL.

### Intestinal volatile fatty acids

Table 5 presents the analysis of intestinal VFA across the different dietary groups. The results show that the DL and LL groups exhibited significantly higher concentrations of propionic acids compared to the C group, indicating a notable effect of the diets on these key VFAs. In addition, formic acid levels were significantly higher in the DL and LL groups while in the same groups lactic acid was lower compared to the C group, further suggesting that the alternative diets influenced the production of certain VFAs in the intestine. In contrast, no significant differences were observed among the diets for other measured VFAs, including citric, succinic, butyric, isobutyric, and valeric acids. This indicates that the inclusion of BSFL did not impact these particular VFAs. Furthermore, the analysis revealed no significant effects of slaughter age on any of the VFAs, nor was there any significant interaction between diet and age for any of the measured parameters. This suggests that the influence of diet on VFA production was consistent across the different ages, and the observed effects were primarily attributable to the dietary treatments rather than the age of the animals or the interaction between these factors.

**Table 5:** The volatile fatty acids (mg/g excreta) in the gut of an indigenous chicken breed fed a diet supplemented with dehydrated black soldier fly larvae and live black soldier fly larvae over the periods 39–147 and 39–174 days of age (means, *n* = 12)

	Diet (D)			Age (A)		SEM	*p*-value		
	C	DL	LL	147 d	174 d		Diet	Age	D x A
Citric	1.132	1.148	0.918	1.154	0.978	0.0707	0.316	0.211	0.247
Succinic	1.662	1.448	0.911	1.39	1.29	0.178	0.201	0.767	0.917
Formic	0.734^a^	0.493^b^	0.388^b^	0.569	0.574	0.0646	0.034	0.964	0.762
Acetic	7.71	8.03	8.12	7.75	7.02	1.320	0.130	0.784	0.258
Propionic	0.752^b^	1.470^a^	1.301^a^	0.901	0.980	0.0301	0.014	0.188	0.589
Butyric	0.696	0.468	0.464	0.528	0.558	0.0520	0.116	0.772	0.444
Isobutyric	5.20	5.73	4.98	4.31	5.03	0.384	0.713	0.210	0.258
Valeric	2.65	3.37	4.54	4.83	4.21	0.523	0.330	0.412	0.325
Lactic	0.825^a^	0.313^b^	0.366^b^	0.660	0.543	0.335	0.039	0.862	0.275
Total	17.7	19.8	18.6	19.3	18.1	1.37	0.672	0.821	0.117

*C *control, *DL *dehydrated larvae, *LL* live larvae, *DxA* interaction diet/age, *SEM* standard error of mean

a, b: *P* < 0.05

### Meat fatty acid profile and atherogenicity and thrombogenicity indices

The fatty acid composition analysis of the breast fillets (Table 6) indicated that the inclusion of BSFL did not significantly affect the overall content of saturated fatty acids (SFA), monounsaturated fatty acids (MUFA), or polyunsaturated fatty acids (PUFA) in the fillets.

**Table 6:** Fatty acids composition (%) and atherogenicity (AI) and thrombogenicity (TI) indices of breast fillet of an autochthonous slow-growing chicken breed fed a basal diet (C) supplemented with dehydrated (DL) or live (LL) black soldier fly larvae slaughtered at 147 and 174 days of age (means, *n* = 12)

	Diet (D)			Age (A)		SEM	*p*-value		
	C	DL	LL	147 d	174 d		Diet	Age	D×A
∑ SFA	31.6	31.9	31.7	31.6	31.4	0.518	0.202	0.254	0.904
∑ MUFA	25.3	25.5	25.1	25.3	24.7	0.396	0.503	0.521	0.446
∑ PUFA	36.7	35.1	35.8	35.9	35.9	0.633	0.230	0.920	0.435
∑ n-6	3.38	3.11	3.26	3.24	3.30	0.118	0.313	0.568	0.621
∑ n-3	33.3	32.0	32.6	32.6	32.6	0.495	0.256	0.988	0.398
∑ n-6/∑ n-3	10.0	10.4	10.2	10.3	10.2	0.258	0.835	0.726	0.570
∑ PUFA/∑ SFA	1.20	1.10	3.31	1.14	2.23	1.130	0.412	0.327	0.401
AI	0.35	0.40	0.37	0.38	0.36	0.065	0.101	0.138	0.795
TI	0.77	0.81	0.81	0.80	0.81	0.048	0.128	0.586	0.919

*C *control, *DL* dehydrated larvae, *LL *live larvae, *DxA *interaction diet/age, *SEM* standard error of the mean of the model, *SFA* saturated fatty acids, *MUFA* mono-unsaturated fatty acids, *PUFA* poly-unsaturated fatty acids, *AME* apparent metabolizable energy, *AI* atherogenicity index, *TI* thrombogenicity index

Moreover, the comparison between experimental groups also showed no significant differences in AI and TI, suggesting that the dietary inclusion of BSFL had no impact on these health-related lipid indices (Table 6).

### Correlation between VFAs, Microbiota, and Cardiovascular risk indices

**Fig. 4:**
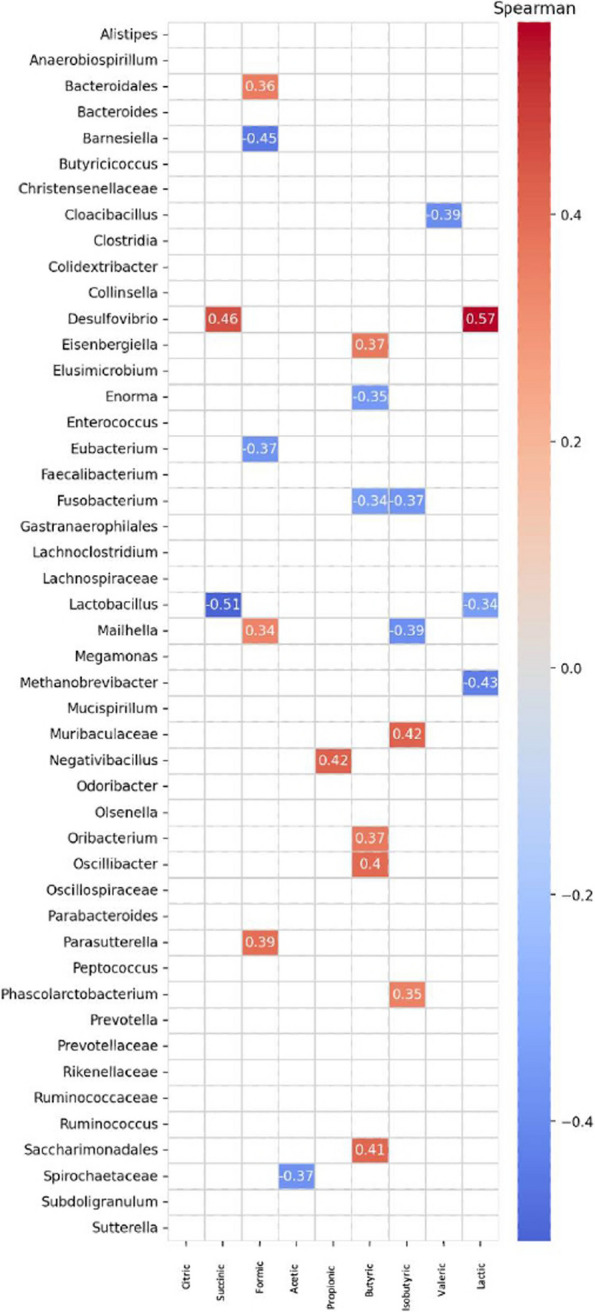
Correlation plot showing Spearman’s correlation between microbial Amplicon Significant Variables (ASV) and Volatile Fatty Acids (VFA) of an indigenous chicken breed fed a diet supplemented with dehydrated black soldier fly larvae and live black soldier fly larvae over the period 39–174 days of age. Only significant associations between ASV and VFA are shown (FDR < 0.05). The intensity of the colors represents the degree of correlation, where blue represents a negative degree of correlation and red represents a positive correlation

**Fig. 5:**
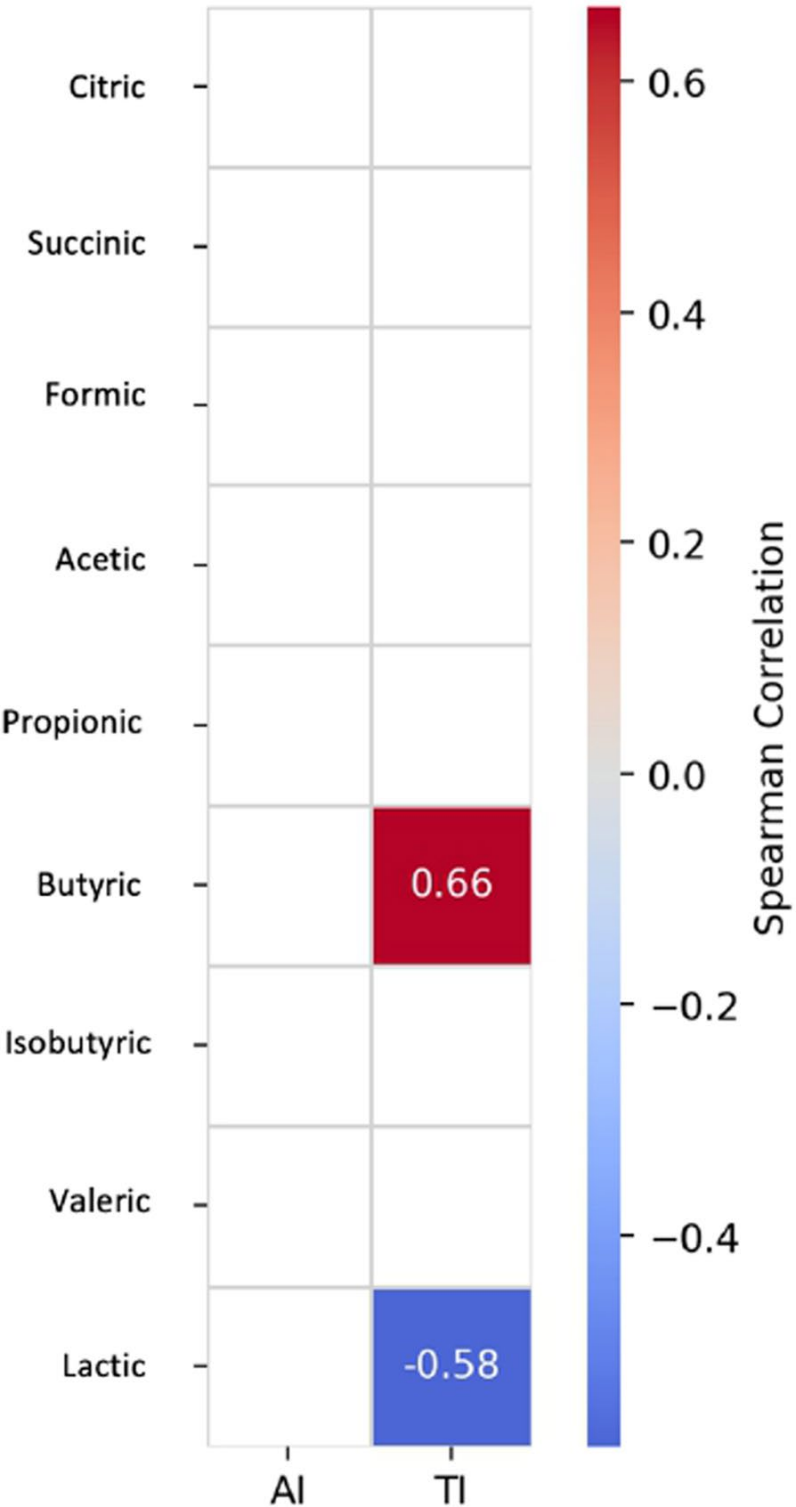
Correlation plot showing Spearman’s correlation between gut microbial Amplicon Significant Variables (ASV) and Atherogenicity (AI) and Thrombogenicity (TI) indices of the breast fillet of an indigenous chicken breed fed a diet supplemented with dehydrated black soldier fly larvae and live black soldier fly larvae over the period 39–174 days of age. Only significant associations between VFA and AI/TI are shown (FDR < 0.05). The intensity of the colors represents the degree of correlation, where blue represents a negative degree of correlation and red represents a positive correlation

Figure 4 presents a correlation plot illustrating Spearman’s correlation between microbial ASVs and VFAs. This plot highlights several notable relationships between specific microbial taxa and VFAs. For instance, the abundance of *Negativibacillus* shows a strong correlation with the production of propionic acid. Additionally, Fig. 5 reveals further correlations between VFAs and cardiovascular risk indices. There is a positive correlation between the production of butyric acid and the trait TI. In contrast, the figure also indicates a negative correlation between the levels of lactic acid and TI, providing additional insights into the associations between these variables.

## Discussion

In this trial the supplementation of BSFL, both dehydrated and live, had a significant impact on the growth performance of a slow-growing indigenous chicken breed. The results obtained show an increase in SW (+ 3.64%) in the BSFL supplemented groups compared to the control group, with results particularly evident in the last month of the experiment. These data are particularly interesting, as they provide new information specific to this native breed, which are often under-explored in poultry research and may help boost the integration between agroecology and the one health approach. A particularly important aspect of this study is the consideration of FCR, a key parameter for evaluating the growth efficiency of birds. In this study, FCR is based on dry matter content. It considers the dry matter from both the feed and the larvae consumed, giving a clearer picture of how BSFL contribute to the diet [21].

Since FCR did not show significant differences compared to the control groups, and consistent with previous research on similar breeds [12, 13, 32], this suggests that BSFL supplementation helped maintain a balance between increased feed consumption and SW. The effectiveness of BSFL in optimizing growth without compromising efficiency is a key advance for the sustainability of poultry feeding. However, the impact of BSFL supplementation is not limited to the growth of chickens, but also extends to meat quality, with particular attention to atherogenic and thrombogenic indices. These indices are crucial parameters to assess cardiovascular risks for meat consumers, especially in a context where concerns about the saturated fat content in the diet is constantly increasing [33]. The growing attention to the quality of fat in meat has led many researchers to investigate how insect supplementation could influence the lipid profile of poultry products. In particular, Kierończyk et al. [34] demonstrated that, although fat derived from BSFL modifies the fatty acid profile in breast muscle, there are no significant changes in atherogenic and thrombogenic indices. This is a particularly reassuring result for consumers, as it demonstrates that BSFL supplementation in chicken diets does not increase the cardiovascular risk associated with poultry meat consumption [35] and it is confirmed by the results of our trial.

Additionally, it is important to consider the effects of BSFL supplementation on the physiological and immune parameters of chickens. Studies have shown that BSFL oil and meal dietary inclusion does not lead to significant changes in intestinal histomorphometry [36] or in inflammatory markers such as IgM, IgA, IgG, and IL-6 [37], which are commonly used to monitor immune health and inflammatory responses in animals [38]. The results of our trial, which align with these previous findings, are particularly encouraging as they indicate that larval supplementation does not pose any inflammatory or immune risks. This makes BSFL, live or dehydrated, a safe and reliable choice for poultry nutrition to obtain a safe and nutritious meat for final consumers. Indeed, some studies even suggest that BSFL oil supplementation may have beneficial effects on intestinal morphology and immune function. For example, Anas et al. [36] found that BSFL oil supplementation, improved the intestinal structure of chickens, increasing the height and width of intestinal villi and strengthening the tight junctions that form the intestinal barrier. This improvement in intestinal morphology is accompanied by a reduction in the levels of pro-inflammatory cytokines, such as IL-6, suggesting a potential anti-inflammatory effect of BSFL. Such improvements in intestinal and immune health could contribute to improved growth performance of chickens, as well as improving their overall welfare.

Hepatic metabolism is another crucial aspect that deserves special attention. In this context, the increase obtained in our trial in the BSFL fed group compared to the control is particularly interesting as it plays a key role in the process of gluconeogenesis, allowing chickens to maintain a correct energy balance, especially under conditions of metabolic stress [39]. Han et al. [40] conducted studies on the purification of FBPase in chicken liver, demonstrating that its activity can be modulated by proteolytic enzymes, such as papain and subtilisin, which improve its stability. The regulation of FBPase is also closely linked to its interaction with other key enzymes, such as phosphofructokinase, suggesting a complex but essential control system for the balance between glycolysis and gluconeogenesis. The presence of an efficient hepatic metabolism is essential to ensure that chickens can respond appropriately to variations in nutrient availability and maintain healthy growth [41].

Furthermore, the role of the gut microbiota in the production of short-chain fatty acids (SCFAs) such as propionic acid and lactic acid is crucial to understand how the diet influence gut health and, consequently, meat quality. Studies such as the one of Liu et al. [42] have shown that increased propionic acid production is associated with the presence of beneficial bacteria such as *Bacteroides* and *Ruminococcus*. These bacteria not only promote the production of SCFAs, but also play an important role in reducing intestinal inflammation, thus improving the overall health of the animals [43]. The results of our study indicate that, although the dietary treatments do not significantly impact overall microbiota diversity, they do influence specific bacterial taxa. The increase in *Faecalibacterium* in the LL treatment, known to produce SCFA, suggests a potential benefit for gut health [44, 45]. The increase in *Colidextribacter* in the DL and LL groups may also have functional implications, although its function is less well understood. These changes point to a more qualitative than quantitative effect, suggesting that microbial composition can be specifically modulated to improve health.

Moreover, the VFA results of our study show that the DL and LL diets significantly increased propionic acid concentrations compared to the C group, indicating a direct influence of these alternative diets on VFA production. This is particularly noteworthy, as propionic acid is often associated with potential benefits for intestinal health [46]. The rise in formic acid and decrease in lactic acid in the same groups further emphasize the diets’ modulatory effects on intestinal VFAs, while other VFAs, such as butyric and citric acids, remained unaffected. Additionally, no significant age-related differences were observed, nor any interactions between diet and age, suggesting that the observed effects are primarily diet-driven. The strong correlation between the abundance of *Negativibacillus* and propionic acid production underscores an intriguing microbiota-VFA relationship. However, the fact that this bacterium was more abundant in the C group compared to the experimental groups raises questions about the effectiveness of the DL and LL diets in influencing specific microbial populations.

Finally, the positive correlation found between butyric acid and TI, indicates that higher butyric acid levels are associated with an increase in TI. Conversely, a negative correlation was observed between lactic acid and TI, suggesting that elevated lactic acid levels are linked to a reduction in TI. These correlations highlight distinct relationships between these acids and the TI, with butyric acid showing a direct association and lactic acid showing an inverse one. Although the underlying mechanisms of these relationships are not yet fully elucidated, it is hypothesized that the presence of lactic acid bacteria in the gut may play a role in enhancing meat quality by modulating the gut microbiota [47]. Lactic acid bacteria are well known not only for their role in promoting gut health but also for their potential to influence the fatty acid composition of meat products. During fermentation, they produce SCFAs, which are vital for maintaining gut health and metabolic balance. This process may contribute to enhanced meat quality, reflecting the broader benefits of lactic acid bacteria on both digestive health and product composition [48, 49]. Additionally, Lactic acid bacteria are involved in the fermentation process of meats, contributing to the development of desirable sensory attributes such as texture, color, and flavor [50]. This modulation of fatty acid profiles may, in turn, contribute to improvements in cardiovascular safety parameters for consumers. Further research is necessary to fully understand the pathways through which lactic acid bacteria exert these effects and their implications for meat quality and health outcomes.

## Conclusion

The results of our study confirm the potential of BSFL administration, both in dehydrated and live form, in optimizing the growth of slow-growing indigenous chicken breeds in an agroecological setting, without compromising feed efficiency or overall animal health parameters considered in this study. In addition to promoting weight gain and improved gut health, BSFL appears to positively affect the gut microbiota and crucial metabolic parameters, such as short-chain fatty acid production, thus improving meat quality. These effects, combined with the absence of risks for consumers’ cardiovascular health, make BSFL a promising supplement for sustainable poultry nutrition. Furthermore, the intermediate slaughter at 147 days revealed potential age-related effects, highlighting how the benefits of BSFL supplementation may evolve with age. However, further studies are needed to fully understand the interactions between diet, microbiota, and meat quality, as well as to explore the potential benefits of BSFL in terms of animal welfare, food safety, and its long-term effects across different ages.

## Data Availability

The authors declare that data are not deposited in an official repository. The data that support the study findings are available upon request.

## Abbreviations

AI: Atherogenicity Index
ASV: Amplicon Sequence Variant
BSFL: Black Soldier Fly Larvae
FAME: Fatty Acid Methyl Esters
FCR: Feed Conversion Ratio
GLM: General Linear Model
RTCC: Ready To Cook Carcass
SCFA: short-chain fatty acids
SEM: Standard Error of Mean
SW: Slaughter Weight
TI: Thrombogenicity Index
VFA: Volatile Fatty Acids
